# The impact of short-duration precipitation events over the historic Cauvery basin: a study on altered water resource patterns and associated threats

**DOI:** 10.1038/s41598-023-41417-6

**Published:** 2023-08-29

**Authors:** Satyajit Ghosh, Dillip Kumar Barik, Parimala Renganayaki, Boosik Kang, Siddharth Gumber, Sundarapandian Venkatesh, Dev Shree Saini, Srichander Akunuri

**Affiliations:** 1 School of Mechanical Engineering, Vellore Institute of Technology, Vellore, 632014 India; 2 School of Earth and Environment, University of Leeds, Leeds, UK; 3 School of Civil Engineering, Vellore Institute of Technology, Vellore, 632014 India; 4 Department of Civil and Environmental Engineering, Dankook University, Yongin, South Korea; 5 Atmosphere, Ice and Climate team, British Antarctic Survey, High Cross, Madingley, Cambridge, CB3 0ET UK; 6 School of Computer Science and Engineering, Vellore Institute of Technology, Vellore, 632014 India

**Keywords:** Environmental sciences, Hydrology, Natural hazards

## Abstract

The Cauvery Delta, the ‘Rice Bowl’ of India follows a time-tested cultivation pattern over several irrigation zones. However, in this era of the Anthropocene, it is now well-established that short-duration, intense precipitation episodes will batter the flood plains year after year. The purpose of this first study is thus to quantify the impacts that such episodes may have on the floodplains of the Cauvery Delta and the concomitant threats to the historic Kallanai Dam. Precipitation events during the North-East monsoon period are driven not just by warm rain microphysics but also by large frozen hydrometeors falling from deep clouds causing undesirable flooding over the region to the extent of 66%. Additionally, from an assessment of the velocity heads and the floodwater depths, this study projects a heightened vulnerability. The total extent of submergence along riverbanks and other flow paths was estimated to be 145.98 ${\mathrm{km}}^{2}$ out of which 65.14% of the submerged area is agricultural land. The most important conceptual advance established in this paper is that sub-zones in major watersheds that are currently safe will get inundated in the RCP8.5 warming scenario in 2050.

## Introduction

South India’s food grain sufficiency is primarily attributed to water resource management in its ‘Rice Bowl district’. For two millennia, the Cauvery Delta has been a glittering example of well-managed irrigation practices with royal patronage bettered with engineering adjustments during the Raj. The Cholan masterpiece (first-second century CE), popularly called the ‘Kallanai Dam’ or the Grand Anaicut, is a gem within the palimpsest landscape of the Cauvery Delta replete with historical significance. The Chola kings were ardent builders and were known all over South Asia. This paper revisits a Cholan masterpiece, i.e., the Kallanai Dam, commissioned by the Cholan King (Rajaraja I) which functions to date regulating water in the Cauvery Delta. It is a water-diversionary structure par excellence and ranks shoulder-to-shoulder with extant structures elsewhere. Kallanai’s ancient foundations consisted simply of sunken unhewed boulders sourced locally and buttressed with double-storeyed structures positioned strategically so that flood waters during both the Southwest and Northeast monsoon periods significantly slow down and spreads over across a spillway to foster paddy cultivation in the catchment subsuming an area of 81,155 ${\mathrm{km}}^{2}$. However, this arrangement might be at risk because of the preponderance of short-duration extreme flood events triggered by global warming. The structure was damaged once before by floods, the veracity of which was hardly ever repeated^1^. However, in the last 5 years, the region was subjected to heavy flooding twice and flood alerts were sounded^2–6^.

There are hardly any studies on the duration and intensity of high precipitation events over the Cauvery Basin. Additionally, studies that have explored the coupling between the microphysical and dynamical processes to provide accurate forecasts of future extremes are limited. A literature citation discourse is provided on two themes, i.e. Inadequacies related to climate modelling studies over Southern India and Inadequacies related to hydrological modelling.

### Inadequacies related to climate modelling studies over Southern India

Seminal studies on rainfall extremes in peninsular India including the works of Roxy et al.^7^, Singh et al.^8^ and Ghosh et al.^9^ have highlighted an increase in the intensity of wet-spells over the region. These studies are useful because they garnered information from rainfall and climate datasets to explain the main synoptic conditions and highlighted the importance of dynamical responses to explain flooding effects. However, they have not investigated the adverse effect of global warming on quantifying water resources over this area. Further, studies on the duration and intensity of high precipitation events over the Cauvery Basin are limited. Goswami et al.^10^ applied palaeohydrological techniques in the upper reaches of the Cauvery basin and found a surge in the rainfall levels causing extreme flood events in this region. Other studies, including the works of Sushant et al.^11^ have only analysed the variability and trends in rainfall over the Cauvery basin during the twentieth century (1901–2002), but have not explained precipitation and flooding patterns in major watershed areas. Extreme precipitation events induced by a warming planet may deleteriously impact paddy and banana cultivation through unduly prolonged submergence of the flood plains with an estimated 68% economic losses over all-natural disaster damages, with farmers often driven to suicides^12,13^.

Only a few studies have explored the impact of human-induced climate change on heritage sites across the world. In a recent study by O’Neill et al.^14^, the authors developed a risk assessment framework combining high-resolution climate model simulations with expert elicitation to garner actionable information that could be used by the relevant stakeholders to safeguard cultural heritage assets. In another study by Kotova et al.^15^, (2023), the authors used an ensemble of climate simulations to analyse variations in climatology for extreme events at two cultural heritage sites in Germany. Although the above study gives projected precipitation intensities for future extreme events, it does not provide a discourse on the vulnerability assessment of heritage structures in the region. There are hardly any studies on the severity and impact of extreme weather events on heritage sites in India mainly influenced by a warmed-up scenario.

### Inadequacies related to hydrological modelling

Flood plain models evolved from lumped models to physically based hydrological models over time^16–18^. Recent, hydrological models have incorporated the use of digital elevation models (DEM), remote sensing, and geographic information systems (GIS)^19,20^. Although, these models can simulate flooding over catchments with few input parameters, they are rather limited in their ability to accrately predict flow velocity which result in significant flooding^21^. Hydrodynamic models may be used as an alternate method of determining flood hydrography, depth, and velocity to make up for these deficiencies^18^. According to Betrie et al.^22^, one of the widely used hydrodynamic models is Hydrologic Engineering Center-River Analysis System (HEC-RAS). There are published papers that predict of extreme rainfall and flood occurrences using the HEC-RAS model. However, only a few studies couple flood risks near dams to local climate change-induced, heavy rainfall events. The purpose of this study was to investigate the potential effects of peak inflows and subsequent flood inundation by coupling a widely used Weather Research and Forecasting (WRF) model with with the HEC-RAS model so that it facilitated a detailing of inundation depths, potential areas of disruption and potential impacts on downstream communities around the Kalanai Dam under two extreme rainfall events—one a recent event in 2022 and secondly, a future warmed event in 2050.

This study is grounded on a real case study of a short-duration intense precipitation event during October 2022. A first-ever climate model run yielded the nature of the precipitation from deep convective clouds over the Kallanai and found that rainfall was mediated equally by warm rain and ice microphysics. Modelled precipitation intensities were used to yield comprehensive hydrological profiling of the Kallanai and Mookumbo regions of the Cauvery delta (Fig. 2) and showed for the first time that the velocity heads in the submerged zones are well above the prescribed threshold and likely to cause dam breaches. This case was contrasted with a future climate scenario, i.e. 2050, when the region is expected to warm further by 1.5 $^\circ \mathrm{C},$ causing even more frequent short-duration intense rainfall episodes. Although extant water resource management practices have facilitated South India’s food grain sufficiency, studies have shown that future atmospheric warming may increase flood risk by altering the distribution and intensity of precipitation events^23–26^. Dams modify the duration and timing of flooding events through water regulation techniques^24^. Therefore, understanding the role of dams in managing short-duration extreme precipitation events in extant and future climate scenarios has become increasingly important. Kallanai can still stand on ancient sunken stones but may require retrofitting to maintain its robust flood-withstanding capacity so that agriculture will continue to flourish as the dam was erected to divert floodwater from the Cauvery into the Kollidam River (Fig. 2) during intense precipitation events when the water level rose above the river’s crest^27,28^. It was reported that during 22–23 August 2018, nine of the forty-five shutters of the Grand Anaicut (another name for the Kallanai Dam) had washed away from a massive deluge, causing a heavy flow of water that concomitantly weakened adjoining piers^5,6^. People along the banks of the Kollidam River were asked to move away following a sudden increase in the water level.

**Figure 1:**
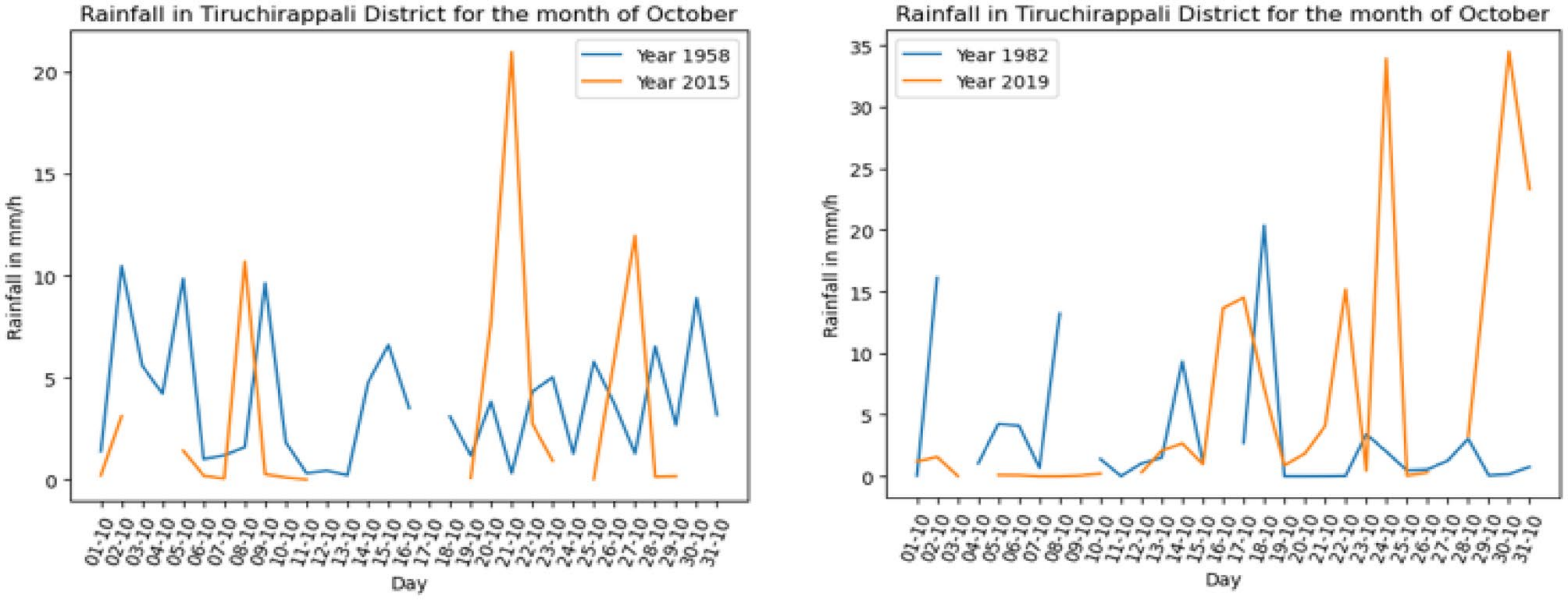
October precipitation amounts obtained from data.gov.in. Meteorological records for Tiruchirapalli District during 1958, 1982, 2015, and 2019. Note intense precipitation over short durations during 2015 and 2019 (created using MATLAB R2022a (Academic License). https://www.mathworks.com).

From an analysis of meteorological records from 1950 onwards, one observes that the frequency of intense precipitation (when more than 20 $\mathrm{mm}/\mathrm{h}$) increased sharply during 2015. Whilst during the 50 s, 60 s, 70 s and 80 s the maximum precipitation happened over several days in a month, during 2015 and in later years the intensities often doubled and were confined to a maximum of two peaks over 30 days. This trend clearly is in sync with the latest IPCC observation that shorter duration yet more intense rain amounts will become the new norm^23^. In Fig. 1, this trend is immediately apparent when one compares precipitation during October for recent years (2015, 2019), showing fewer distinct peaks with almost double the rain amounts compared with data from the same month (1958, 1982).

**Figure 2:**
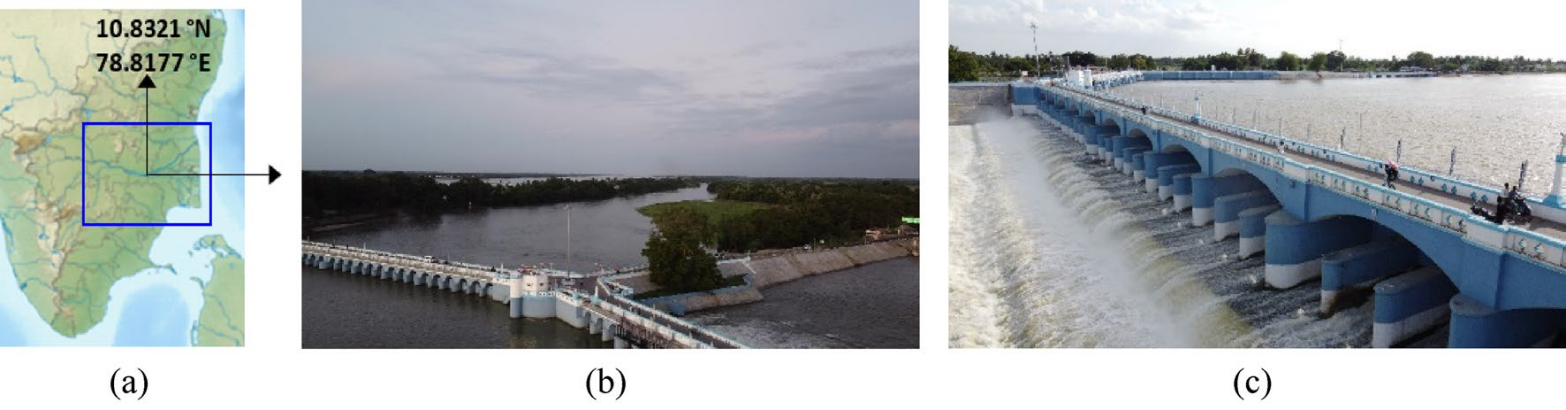
(**a**) Google Map image of peninsular India showing the zoomed-in view of the Kaveri Basin enclosed in a blue box (created using Origin Pro 2023b (Learning Edition) URL: https://www.originlab.com/). (**b**) Aerial view of the Kallanai dam and the watershed region below it shot from a camera mounted on a multi-rotor drone. (**c**) A closer view of the Kallanai (Photos: Authors).

What is currently called the Mukkombu (or the Upper Anaicut) is a 685 $\mathrm{m}$ long structure built more recently during the nineteenth century by Sir Arthur Cotton and is 30 $\mathrm{kms}$ away from the 2nd Century Kallanai Dam built during the Chola period. The island of Srirangam is close to the Upper Anaicut between the Thanjavur delta, with hundreds of villages facing imminent threats increasingly frequently. See Fig. 2 showing a map marking Kallanai, Mukkambu, Kaveri, Kollidam, and Srirangam and supplementary Drone Videography (S1) showing an overview of the upstream and downstream parts of the Kallanai Dam.

The preceding descriptions testify that as extreme but short-duration precipitation events become the new norm, the fragile ecosystem nurtured by the ancient Cholans two millennia ago is becoming increasingly vulnerable because of recurrent flood threats. With several shutters getting washed away from a monsoon rain event in the Upper Anaicut in the past, it is uncertain to what extent the iconic and ancient Kallanai Dam will withstand flood furies and continue to serve as a flood water regulator along Kaveri’s fertile plains.

An earlier study by Goswami and co-authors explored major flood events in the upper Kaveri basin from high-intensity short-duration storms through *dating* techniques^29^. Gupta and Dhanya^30^ have relied on satellite-derived information along with rainfall and temperature data to estimate flood potential in parts of the Kaveri basin. However, to date, there has been no study to characterise the microphysical attributes causing such deluges over this region, nor have there been any hydrological modelling of present and future scenarios to explore quantitatively the most vulnerable areas comprising of irrigation lands and human habitats and notably also the most vulnerable parts of the Upper Anaicut and the historic Kallanai.

The main objectives of this study are:

- To understand the synoptic and microphysical characterisation of a recent short-duration heavy precipitation event during October 2022 and ascertain the main processes (warm rain alone or along with ice microphysics) involved. This separation is crucial because it has been recently found that ice processes yield giant raindrops with higher impaction velocities^31–33^.
- What was the depth of flood water over upstream and downstream regions along both dams and how do they compare with government-prescribed thresholds?
- Finally, to what extent is there a crop damage threat, and where are the most vulnerable areas?

Figure 2b shows the aerial view of the Kallanai dam and the surrounding area shot from varying stand-off distances using a camera mounted on a multi-rotor drone. From Fig. 2b, one also notices upstream flooding mediated by water level rise, submerging agrarian land on either side of the reservoir.

The study region in question received intense rainfall during October 2022. Flood alerts were sounded along the Kollidam River, a northern distributary of the Cauvery River (see Fig. 2b;^2,3^). The overflowing rivers inundated the paddy and banana crops on hundreds of acres in Panayapuram, Uthamarseeli and Kilikoodu along the Kallanai Trichy road and intruded into the streets of Nadhalpadugai, Mudhalaimeduthittu and Vellamanal near the Kollidam stretch (see Fig. 2b;^2,3^).

The scheme of the paper is as follows:

- A high-intensity precipitation event (5^th^ of October 2022) that caused catastrophic flooding over the study region is first described with a complete meteorological discourse on the prevailing synoptic conditions.
- The observed cloud morphology is compared with results from a full-scale numerical weather prediction model, which includes a double-moment microphysical scheme with nine prognostic variables for five classes of hydrometeors. This is followed by validating the modelled microphysical attributes with satellite-derived observations.
- A GIS-based Digital Elevation Model (DEM) is used to demarcate sub-watersheds in the Cauvery Delta. Hydrological parameters, including the curve numbers, are ascertained to quantify storm-water runoff potentials in each sub-watershed.
- Model-derived precipitation intensities and digital elevation information are used to configure a GIS-based hydrological model to predict flood depth and discharge velocities in the basin in the 2022 and 2050 scenarios with an assessment of threats and vulnerabilities related to irrigation and human settlements.

Studies have only qualitatively talked about the failure of the dam structures in general without linking it to Climate Change threats in a warming scenario. Additionally, studies on the detailed coupling of climate models with hydrological models are scanty. The present paper overcomes this lack of rigorous quantification of projected risks through a three-tiered protocol. First, a GIS-based digital elevation model was used to quantify topographical variations, land-use patterns, and soil maps. Secondly, we have used a time-tested advanced high-resolution climate model over the Cauvery Delta. A rigorous microphysical detailing using a double-moment scheme (wherein the hydrometeor mixing ratios as well as the number counts are factored in) is used for a full microphysical characterisation. Thirdly, hourly outputs of rain amounts are coupled to a robust hydrological model to map the floodplains spanning a covered area of 7861 ${\mathrm{km}}^{2}$. This three-tier downscaling is not only utterly novel, but also the fullest possible modelling prototype for a definitive quantification of floodwaters in the Cauvery Delta in extant and future warmed-up scenarios. This procedural detailing addresses the large Environmental significances as well and are described as a brief section sub-head:

### Environmental significance

The environmental significance of such a study is enormous. Not only is it timely but is also definitive because it has provided details of the impact of a recent, short-duration, intense precipitation event addressing the relative contribution of several hydrometeor class (ice, snow, graupel, and liquid cloud) over the Cauvery Delta. Such short-duration events cause flooding over upstream as well as downstream regions of a major water-diversionary structure. This impacts paddy and banana cultivation and the safety of low-lying inhabited tenements. What obtains now is likely to be greatly exacerbated over the next two decades. One must estimate the speed of flowing water in the inundated regions over areas close to dam sites, in telling details because this can erode agricultural land resulting in soil loss and decreased fertility. Further, such high-velocity water flow can inflict physical damage to structures, particularly those positioned close to the riverbanks.

## Materials and methods

**Figure 3:**
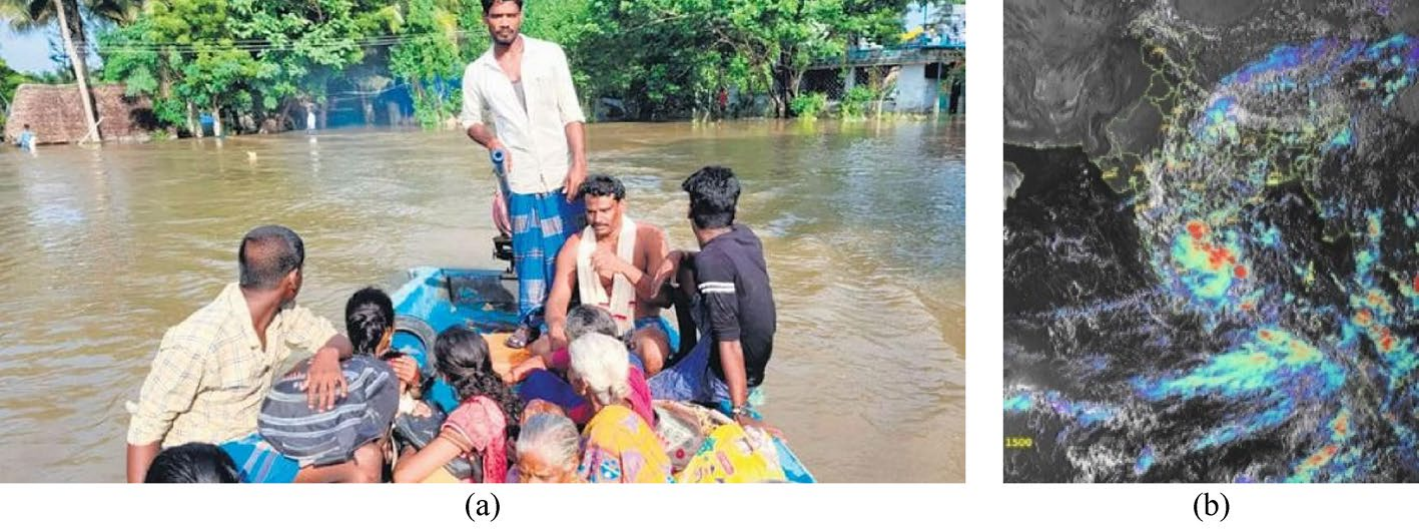
(**a**) Submerged regions in the delta region during the October 2022 floods $$^\circ \mathrm{N}$$$$^\circ \mathrm{E}$$ (Source: https://www.newindianexpress.com/states/tamil-nadu/2022/oct/19/kollidam-block-villages-in-mayiladuthurai-hit-by-flood-for-fifth-time-this-year-2509571.html)^2^. (**b**) Cloud cover over the southern province of India on the 05th of October 2022 0930 IST. Note the extensive cloud coverage over the Trichy district (10.7905 °N, 78.7047 °E) (Source: Meteorological & Oceanographic Satellite Data Archival Centre, Space Applications Centre, ISRO. https://www.mosdac.gov.in/).

The synoptic conditions during this period were characterised by north-westerly low-pressure troughs, which eventually deluged the district of Tiruchirappalli and other neighbouring regions (Fig. 3a). Figure 3b shows the cloud cover over peninsular India on the 05th of October 2022, 1500, IST (Source: Meteorological & Oceanographic Satellite Data Archival Centre, Space Applications Centre, ISRO).

**Figure 4:**
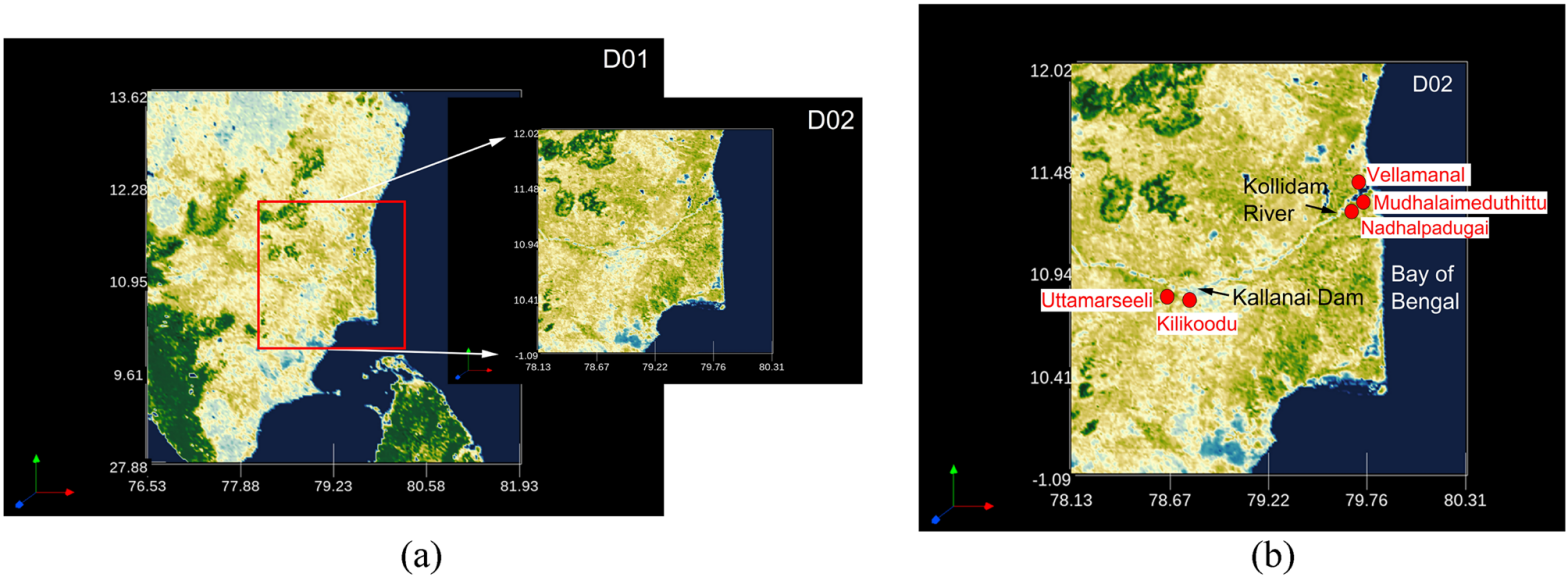
(**a**) Outer and Inner domains for WRF simulations. The outer domain (D01) covers the spatial extent of 597 $\mathrm{km}\times$ 597 $\mathrm{km}$ with a resolution of 3 $\mathrm{km}$, and subsumes a finer inner domain covering an area of 240 $\mathrm{km}$ $\times$ 240 $\mathrm{km}$ with a resolution of 1 $\mathrm{km}$. (**b**) A zoomed-in inner domain with the vulnerable villages identified in red. Also, note the positioning of the Kallanai Dam and Kollidam on the map (created using Origin Pro 2023b (Learning Edition) https://www.originlab.com/ and VAPOR: A Visualization Package Tailored to Analyze Simulation Data in Earth System Science v 3.6.0 http://www.vapor.ucar.edu/).

The associated cloud system is modelled with the Weather Research and Forecasting (WRF) model^34^. The WRF model (version: 3.8.1) was configured to run with two domains, i.e. an inner, nested domain with *finer* resolution (1 $\times$ 1 $\mathrm{km}$) and spatial extent of 240 $\mathrm{km}$ $\times$ 240 $\mathrm{km}$, covering the Kallanai dam and Kollidam regions, and embedded within a coarser-resolution (parent) (3 $\mathrm{km}$) outer domain with an area of 597 $\mathrm{km}$ $\times$ 597 $\mathrm{km}$ covering the entire southern peninsular India (see Fig. 4).

Model simulations are conducted for two scenarios, i.e., (1) A real scenario of a precipitation event spanning 2nd–6th October 2022 over the study region. (2) A future October 2050 run focused on a warming scenario, also characterised by high emissions, which is a likely outcome if concerted efforts are not made to cut down emissions. Real-time meteorological data (NCEP FNL data 1° × 1°) for the ‘real case’ required for initialising the model were retrieved from the research data archive of the Computational and Information Systems Lab, National Centre for Atmospheric Research (NCAR). The above dataset is prepared operationally from the Global Data Assimilation System (GDAS), every 6 h (https://rda.ucar.edu/datasets/ds083.2/). The simulation progressed from the 2nd of October 2022, 0000 $h$ UTC (0530 IST) to the 6th of October 2022, 1800, $\mathrm{h}$ UTC (2350 IST), when distinct cloud activity was observed. The model set-up comprised 50 vertical levels and was subsequently integrated with a time step of 6 $\mathrm{s}$.

Meteorological variables for initialising the WRF model for the future warming case have been sourced from the NCAR’s Community Earth System Model (CESM), which participated in phase V of the Coupled Model Intercomparison Experiment (CMIP5) and supported the Intergovernmental Panel on Climate Change Fifth Assessment Report (IPCC AR5)^35^. The variables available at six hourly intervals are interpolated over 26 pressure levels and are bias corrected using the European Centre for Medium-Range Weather Forecasts (ECMWF) Interim Reanalysis (ERA-Interim)^36,37^. Files are available for three Representative Concentration Pathway (RCP) future scenarios (RCP4.5, RCP6.0 and RCP8.5) spanning 2006–2100. In the present study, we have only considered the RCP8.5 case, i.e. ‘high-emissions scenario’, which is a likely outcome if society does not make strenuous efforts to cut emissions.

Cumulus convection was modelled only in the outer domain through the widely used Kain–Fritsch scheme based on the mass flux approach for shallow and deep clouds, whilst this option was not employed for the cloud-resolving inner domain^38^. The vertical structure of the boundary layer was resolved through MYNN 2.5 level TKE scheme planetary boundary layer (PBL)^39^. In addition, atmospheric heating from incoming shortwave and outgoing longwave radiations was quantified using the rapid radiative transfer model schemes. To account for the mass and the number concentration of the hydrometeors, Morrison's double-moment microphysics scheme within the remit of the WRF model was used, which features five classes of hydrometeor namely liquid cloud, ice, snow, graupel and rain^40^.

**Figure 5:**
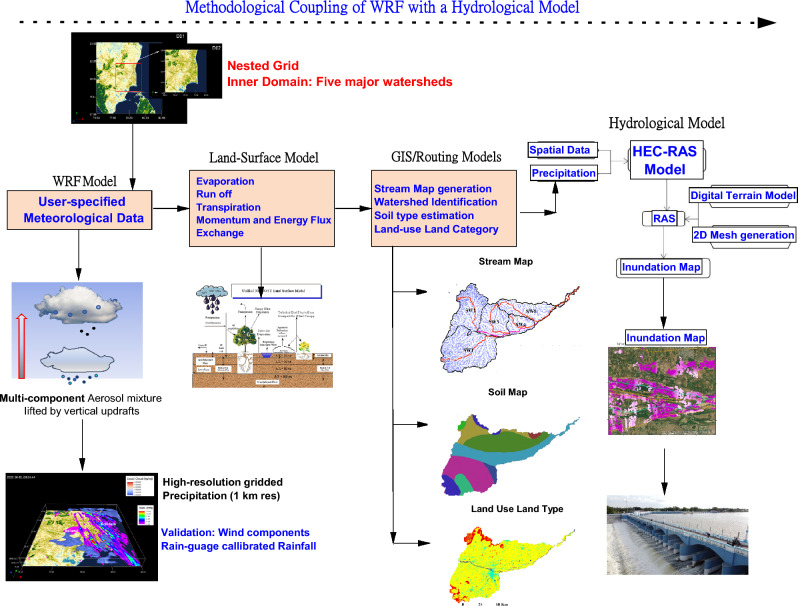
Schematic diagram showing the methodological coupling of WRF model outputs to a terrestrial hydrological code, i.e. HEC-RAS (Hydrologic Engineering Center’s River Analysis System). Land use, soil map, soil moisture, terrain data and precipitation characteristics are some of the primary inputs to the HEC-RAS model (created using Origin Pro 2023b (Learning Edition). https://www.originlab.com/).

To examine the extent of submergence of vulnerable areas and undertake impact assessment studies vis-à-vis extreme precipitation events, a coupling framework is required to link multi-scale atmospheric model outputs to terrestrial hydrology enabling a reliable streamflow prediction across basins and sub-basins. A methodological coupling between climate model outputs with a hydrological code is shown in Fig. 5.

Additional details on the nature of the coupling between the modelling suites used in this analysis are provided in the subsequent sections.

### GIS modelling

Quantifying the land use and land cover (LULC) profile was essential to closely delineate cropped and uncropped regions. LULC map for the study area basin was prepared to indicate five land categories, including water bodies, vegetation cover, agricultural land, built-up area, and barren/scrubland. This was done by importing the ESRI (Environmental Systems Research Institute, Inc.) LULC map in the ArcGIS environment and then using Satellite-based Remote sensing procedures involving the use of Landsat-8 Operational Land Imager (OLI) images retrieved from the United States Geological Survey (USGS)^41^. LULC maps were developed from high-resolution (10 $\mathrm{m}$ spatial resolution) multi-spectral Geo TIFF images covering seven visible and near-infrared (VNIR) wavelength bands ranging between 0.43 and 2.29 $\mathrm{\mu m}$ using ArcGIS Pro^42,43^. The first step involved stacking the retrieved multi-spectral images to form a composite multi-band raster for subsequent downstream analysis. A supervised classification algorithm was used to obtain relevant training samples to classify images based on associated spectral signatures.

**Figure 6:**
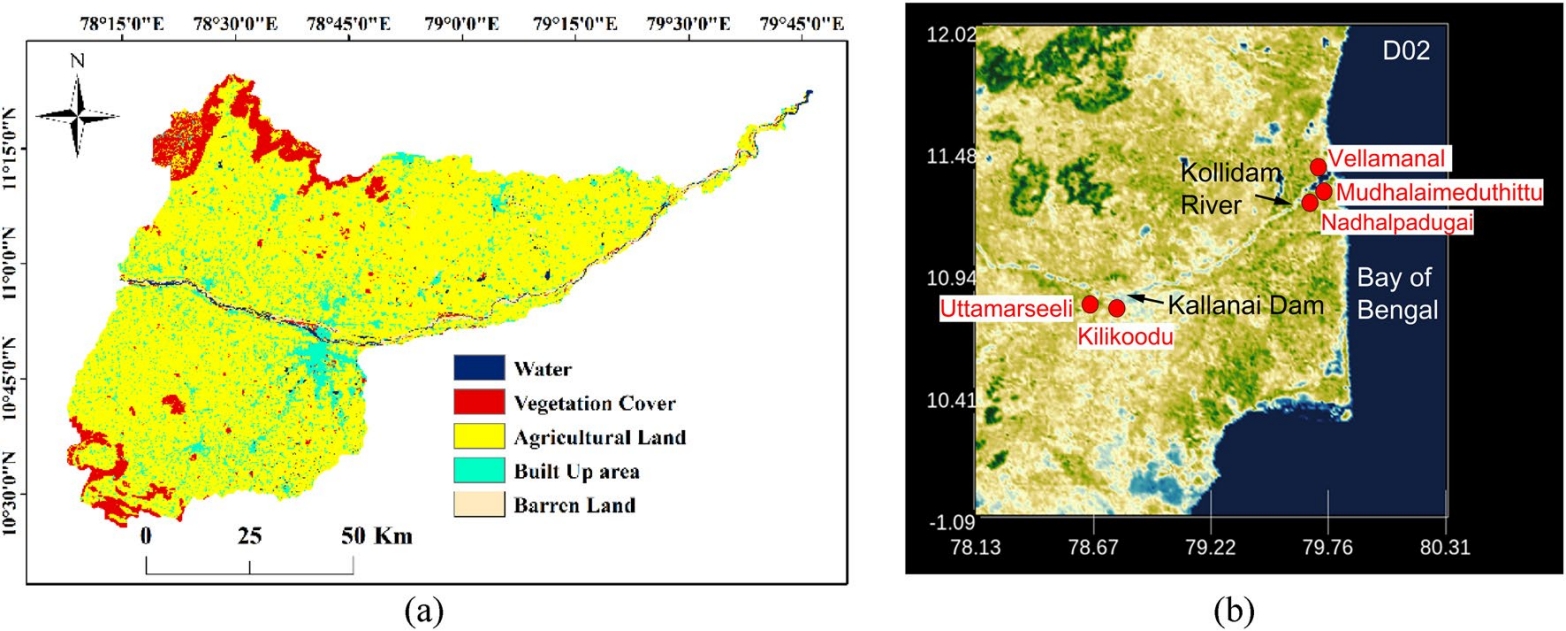
(**a**) GIS-based Landuse Landcover characterisation indicates a significant stretch of agricultural land in the delta region (created using ArcGIS, Esri^42^ https://www.arcgis.com/index.html). (**b**) Map showing the positioning of the Kallanai dam, Kollidam River and vulnerable areas, including villages at risk of inundation from heavy precipitation events (created using Origin Pro 2023b (Learning Edition) https://www.originlab.com/ and VAPOR: A Visualization Package Tailored to Analyze Simulation Data in Earth System Science v 3.6.0 http://www.vapor.ucar.edu/).

**Figure 7:**
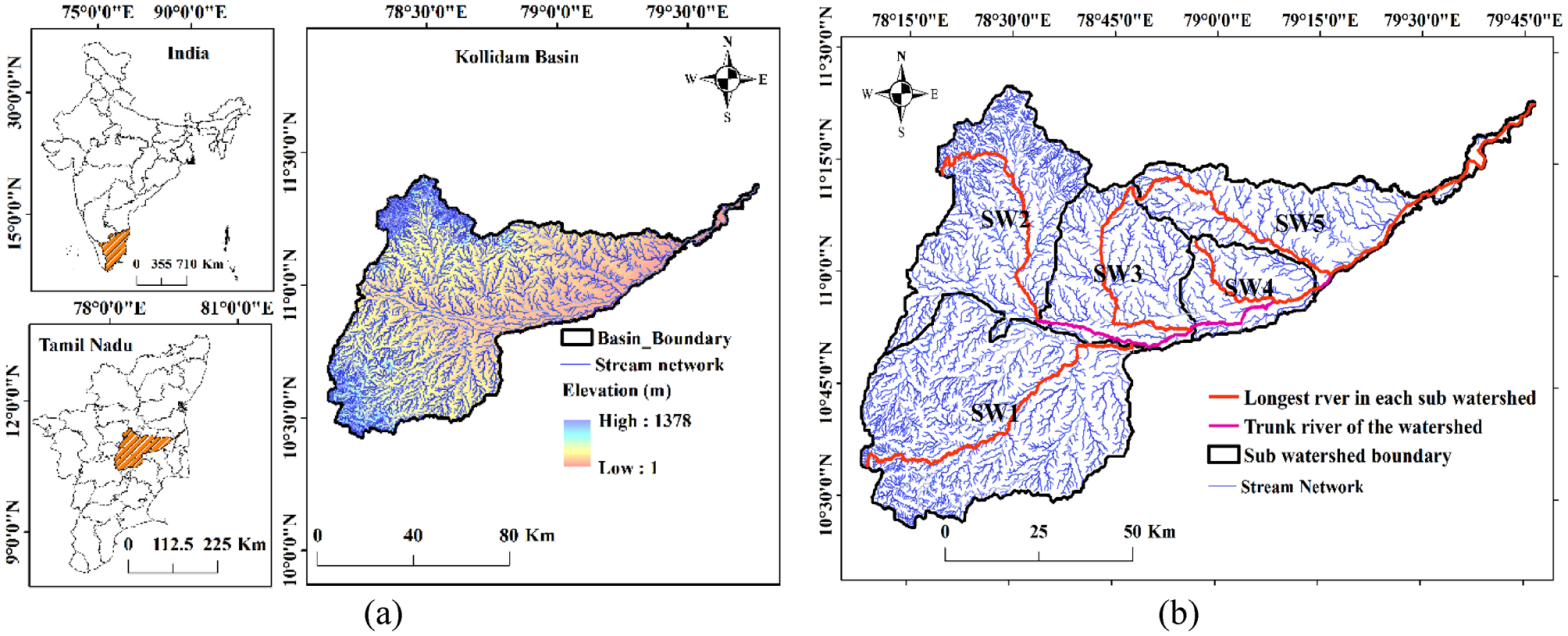
(**a**) Map of India showing the chosen region and the corresponding Digital Elevation Model (DEM) developed in the ArcGIS environment. (**b**) Stream order and sub-watersheds in the Cauvery Delta. The longest and the trunk rivers in each sub-watershed are identified in red and pink lines, respectively (created using ArcGIS, Esri^42^ https://www.arcgis.com/index.html).

It is clear from Fig. 6 that regions surrounding the flowing Cauvery (in blue- indicating ‘water’) mainly include agricultural lands (in yellow), and through this study, we aim to demarcate vulnerable regions which are at severe risk of inundation during extreme precipitation events. The next step was delineating sub-watersheds in the study area to ascertain flow direction and accumulation across zones. This required downloading the Digital Elevation Model (DEM) from USGS earth explorer in the Tag Image File Format (TIFF). To develop the elevation model for the chosen study region, four TIFF files were deemed sufficient that covered the entire chosen stretch. Further, a mosaic of the whole section was developed with the help of the ‘mosaic to new raster’ option available within the ‘data management module’. Finally, the DEM image of the entire basin was generated after removing sinks and peaks using the ‘Spatial Analyst Tool’. It was found that the elevation in the study area varies from 1 to 1378 $\mathrm{m}$. The DEM enabled an examination of the flow direction and accumulation within the basin and sub-basins, further aiding in the construction of a stream raster map using the STRAHLER stream order method. The stream map was then superimposed onto five sub-watersheds (SW1–SW5) to get a handle on the routing map in the sub-basins and this is shown in Fig. 7.

The mean elevation, total area and length of the longest river in each sub-watershed are, respectively, SW1 (489.63 $\mathrm{m}$, 2901.4149 ${\mathrm{km}}^{2}$, 107,526 $\mathrm{m}$), SW2 (726.5 $\mathrm{m}$, 1750.8247 ${\mathrm{km}}^{2}$, 83,404.9 $\mathrm{m}$), SW3 (311.717 $\mathrm{m}$, 1254.8 ${\mathrm{km}}^{2}$, 73,238.7 $\mathrm{m}$), SW4 (74.5 $\mathrm{m}$, 636.16298 ${\mathrm{km}}^{2}$, 52,376.8 $\mathrm{m}$), SW5 (256.64 $\mathrm{m}$, 1318.6811 ${\mathrm{km}}^{2}$, 163,769 $\mathrm{m}$).

**Figure 8:**
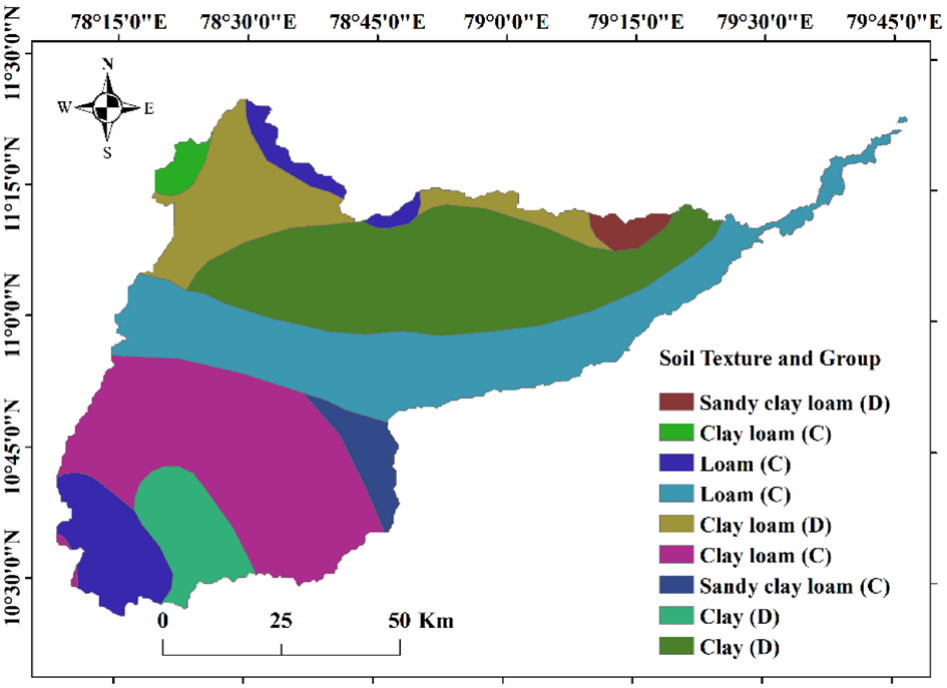
Soil profile in the Cauvery Delta (created using ArcGIS, Esri^42^. https://www.arcgis.com/index.html).

The above-mentioned GIS-based remote sensing procedures for determining land use, soil type, soil moisture, and the region’s elevation profile were necessary to estimate the ‘curve number (CN)’, which is a hydrological parameter used to examine the storm-water runoff potential in drainage areas. The associated soil map required for estimating the CNs is prepared using the Food and Agricultural Organization (FAO) soil database (https://www.fao.org/soils-portal/data-hub/soil-maps-and-databases/faounesco-soil-map-of-the-world/en/) and is shown in Fig. 8.

It is interesting to note that the land type surrounding the flowing Cauvery mainly constitutes loamy soil, which fosters paddy and banana cultivation in the region (Fig. 8).

With a GIS-based assessment of sub-watersheds in the Cauvery Delta and a quantification of precipitation intensities from a sophisticated CFD model run, it is possible to generate flood maps to examine regional and local flooding in the region. Rain rates are quantified by a sophisticated double-moment microphysical scheme within the remit of the widely used Weather Research and Forecasting (WRF) model, which also accounts for the number concentration of hydrometeors. Modelled microphysical and dynamical outputs have been suitably benchmarked with real-time satellite observations to highlight the robustness of the modelling methodology and these results are discussed in the “Results and Discussion” section. We shall now describe the characterisation of the Hydrological code that used inputs from the WRF model to yield contours of flooding along with velocity profiles.

### Hydrological modelling

Flood inundation maps are useful tools for assessing flood risks and highlighting evacuation routes and emergency shelters for quick disaster relief during flood alerts. We have resorted to the Hydrologic Engineering Centre’s River Analysis System (HEC-RAS), an open-source hydraulic model to create a 2-D flood inundation map over the Kaveri delta and estimate flow velocities of flood waters during this heavy rainfall event^44^. The hydrological and topographical data are the model’s main inputs. Topographic data from a DEM along with the appropriate projection files were loaded to initiate the mapping of flood-prone areas, with the boundary conditions defined by creating vector polylines of hydrological entities such as rivers, flow paths, and banks. Cross-section lines perpendicular to the river, the riverbank, as well as along the flow path were included at regular intervals. Hydraulic parameters such as the normal depth of upstream and downstream portions, as well as Manning’s Roughness Coefficient along the right and left banks, and along the course of the river were estimated from the surrounding land cover. The predicted intensities of precipitation ascertained from the WRF model (described earlier) yielded the peak discharges in each sub-watershed and were used as inputs to the HEC-RAS model to demarcate flood-prone areas. This is the first study that couples the WRF with the HEC-RAS model applied over this strategic region housing an iconic heritage structure, a region that also promotes irrigation to yield paddy to feed millions. The simulations yielded a steady flow so that the surface elevation and velocity of flow could be ascertained. The results were then visualised also within the HEC-RAS framework with well-marked depth and velocity profiles along with flood inundation maps. These procedural attributes are indicated in Fig. S2. The first step in this methodological framework was to ensure that, with the above specifications, we could get all the broad attributes right to proceed with further small-scale configurations.

## Results and discussions

**Figure 9:**
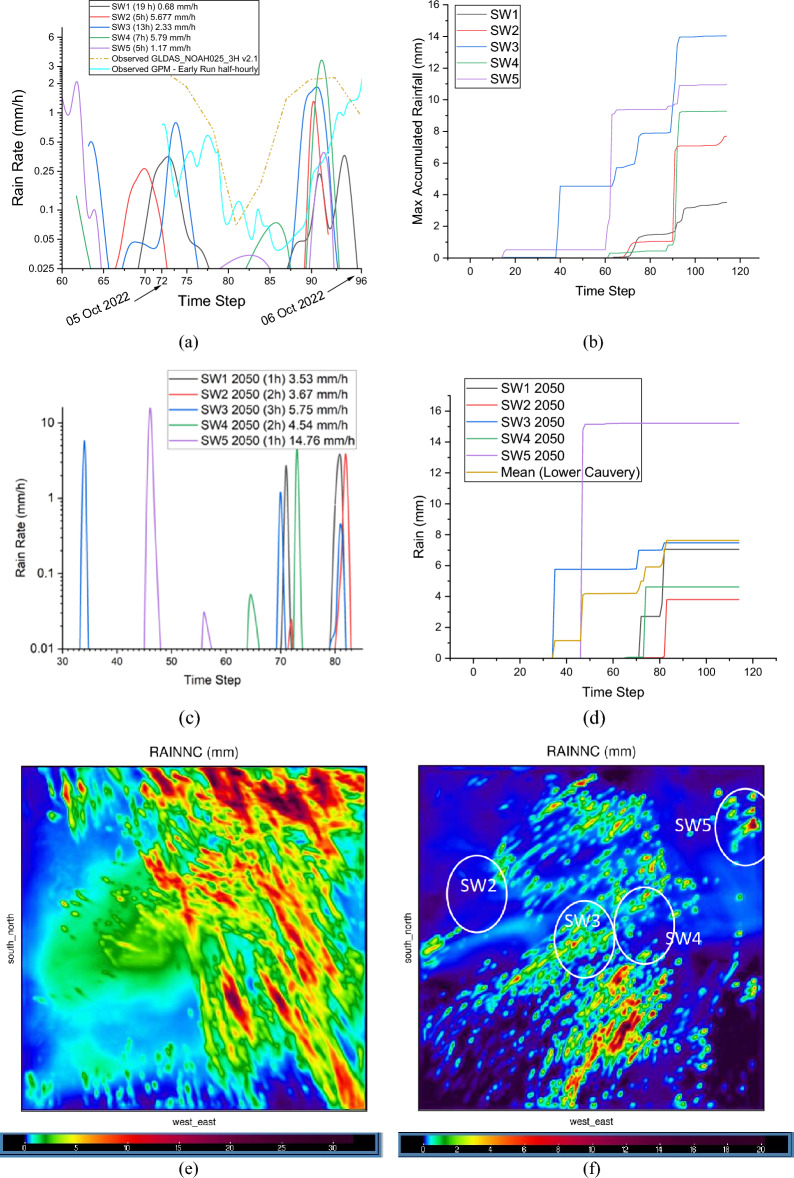
(**a**) Modelled and Observed rain rates in sub-watersheds during the 5th and 6th of October 2022 ($\mathrm{mm}/\mathrm{h}$). Note that the model configured with Morrison’s double moment scheme is able to reasonably reproduce diurnal variations in rain rates over the study region during the chosen time window. Area-averaged observed rain rates have been sourced from the 3-hourly GLDAS Model product (GLDAS_NOAH025_3H v2.1) over the selected region 78.4277 E, 10.1045 N, 80.2954 E, 12.0161 N. (**b**) Modelled accumulated rainfall in sub-watersheds during 5th and 6th of October 2022. (**c**) Modelled rain rates ($\mathrm{mm}/\mathrm{h}$) in sub-watersheds during October 2050. Note short-duration (1–3 $\mathrm{h}$) increased rainfall events in SW1, SW3 and SW5. (**d**) Modelled accumulated rainfall in sub-watersheds during the 5th and 6th of October 2050. (**e**) Spatial distribution of accumulated received precipitation ($\mathrm{mm}$) over the chosen study region (inner domain) during 2nd–6th October 2022. (**f**) Spatial distribution of accumulated received precipitation ($\mathrm{mm}$) over the chosen study region (inner domain) during October 2050 (created using MATLAB R2022a (Academic License) https://www.mathworks.com and Origin Pro 2023b (Learning Edition) https://www.originlab.com/).

The high-resolution Weather Research and Forecasting (WRF) simulations yielded a detailed insight into the evolution of rain rates between the 5th and 6th of October 2022 and for a future 2050 scenario. Figure 9a shows the temporal profile of rain rates in each sub-watershed (from SW1 to SW5) based on the maximum accumulated rainfall in each zone. One notices that the maximum rain rate of 5.79 $\mathrm{mm}/\mathrm{h}$ is noted in SW4 (green line), and the total rainfall duration in this basin between the 5th and 6th October 2022 is 7 $\mathrm{h}$ (see the bracketed quantity in the legend). The rainfall duration can be estimated from the figure by counting the incremental time steps under the curve, where each timestep corresponds to 1 $\mathrm{h}$. For instance, in SW4, rainfall was noted between timesteps 74–75, 87–88, and 89–94, amounting to 7 timesteps equalling 7 $\mathrm{h}$ of rain. The maximum rain rate and the rainfall duration in each sub-watershed are provided in the legend. One also notices that modelled rain rates in the chosen time window agree favourably with area-averaged observations sourced from the 3-hourly GLDAS Model product (GLDAS_NOAH025_3H v2.1) over the selected region (orange dash-dot line). In fact, the modelled precipitation values approaching 2.5 $\mathrm{mm}/\mathrm{h}$ also agree with the rain-gauge calibrated multi-satellite precipitation estimates shown by the cyan line plot in Fig. 9a.

**Figure 10:**
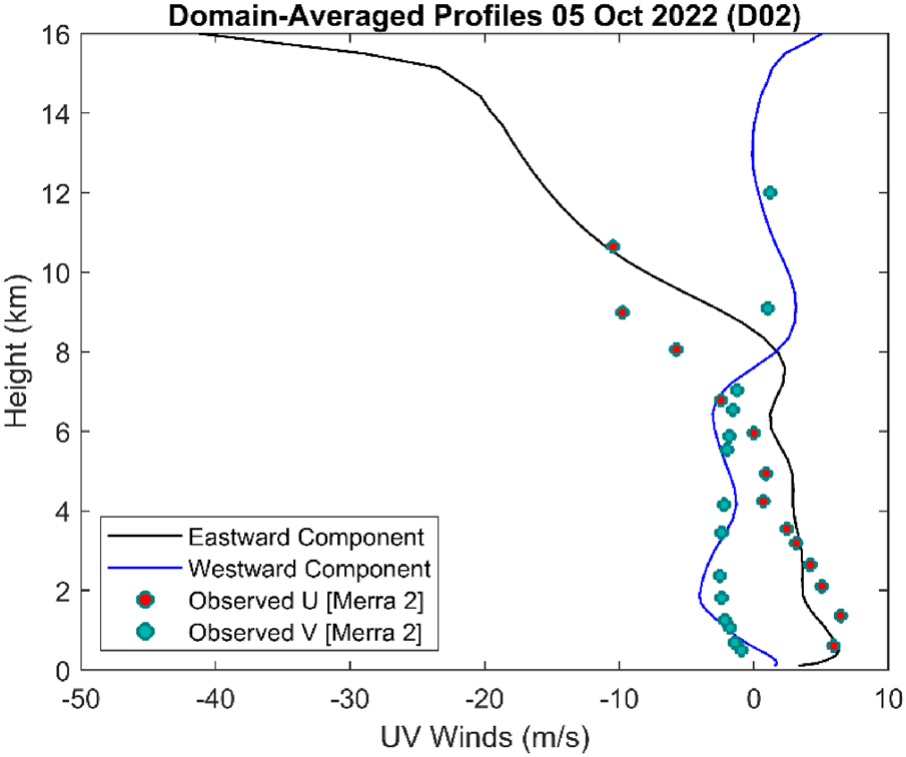
Modelled and observed wind profiles in the inner domain. Note that the modelled wind components (solid lines) agree favourably with the observed values (scatter points) (created using MATLAB R2022a (Academic License) https://www.mathworks.com).

Not only are the modelled rain rates validated with satellite-derived observations for the 2022 scenario, but we have also compared dynamical wind components from the WRF model with MERRA-2 observations and found a reasonable agreement (see Fig. 10).

Figure 9b shows the temporal profile of the maximum accumulated rainfall in each zone. One notices that the maximum rain depth of 14 $\mathrm{mm}$ is noted in SW 4 (green line), where the maximum rain rate was recorded. Pour Point 1 is in SW1, where only 3 $\mathrm{mm}$ of rainfall was observed.

We also show precipitation intensities and accumulated rainfall depth for a future rainfall event in October 2050 based on the highest emissions scenario, i.e. RCP8.5 (Fig. 9c and d). Figure 9c typifies short-duration (1–3 $\mathrm{h}$) rainfall extremes over the study region corroborating published results linked to the intensification of short-duration rainfall excesses in future scenarios^45^. It is interesting to note that the maximum rainfall rate along both the Kallanai and the Mokkambu plains (SW3) approaches 5.75 $\mathrm{mm}/\mathrm{h}$ in the 2050 case (blue line plot in Fig. 9c) compared to a 59.4% lower value of 2.33 $\mathrm{mm}/\mathrm{h}$ received in the 2022 run (blue line plot in Fig. 9a). As we show later, such moderate to heavy rainfall rates have a greater tendency to cause local floodwater inundation, which may inflict physical damage to structures, particularly those positioned close to the riverbanks. At this stage, the spatial distribution of accumulated precipitation in the two scenarios must also be discussed. SW1, SW3, and SW5 encounter increased precipitation intensities that are more localised in the later 2050 run, with other sub-watersheds, i.e. SW2 and SW4 recording reduced rainfall rates by up to 35.3% and 21.5%, respectively (Fig. 9e and f). Such differentiated rainfall intensities over short durations across sub-watersheds may cause rapid submergence of agricultural lands adjoining the Kaveri River belt, and this may have non-intuitive hydrological implications, which will be subsequently discussed.

We now discuss the nature of the precipitation from deep convective clouds over the study region. It is observed from Fig. S3 that the inner domain (ID) centred around the Kallanai region contained more cloud and rain amounts (solid line plots) than those observed in the outer domain (OD). One also notices that the rain mass increases with a sharp dip in cloud mass amounts, owing to efficient auto-conversion and accretion processes. At this stage, discussing the vertical profiles of domain-averaged hydrometeor mixing ratios and the embedded temperature structure is essential. Figure S3a distinguishes between the warm and cold regions of the observed cloud, separated by a 0 °C isotherm (~ 4.5 $\mathrm{km}$). A clear cloud base at 1 $\mathrm{km}$ is observed in the inner domain (solid black line). Liquid cloud (marked in black line plots) spans a vertical range between 1 and 8 $\mathrm{km}$, where warm rain microphysics prevails over regions positioned below 5 $\mathrm{km}$ and cold cloud processes operate further aloft. Other cold hydrometeors, including graupel, ice and snow, also contributed to the observed rainfall amounts.

**Figure 11:**
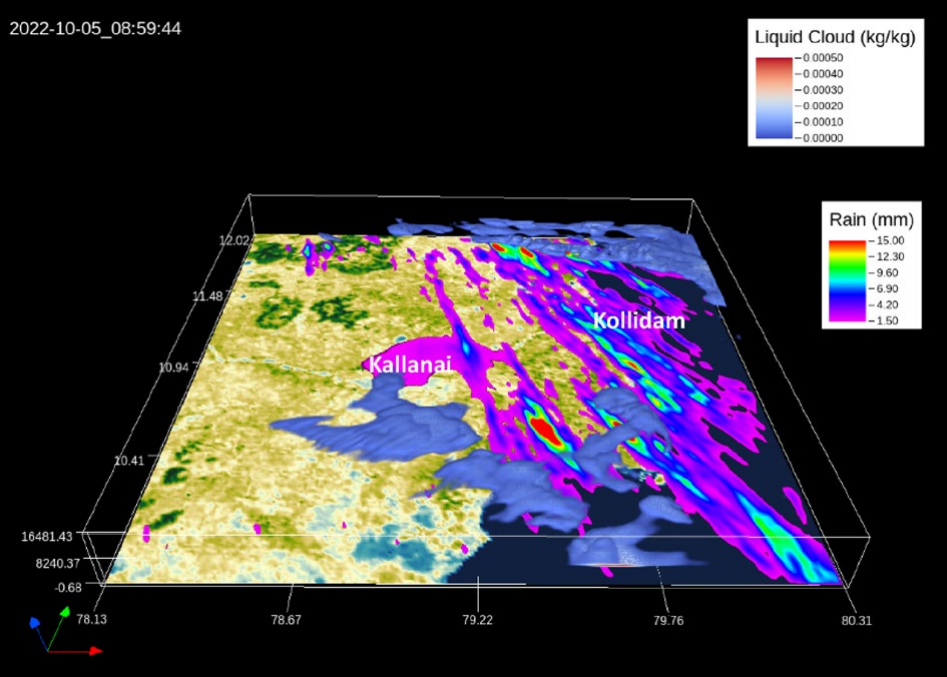
A 3D-rendered image showing cloud volume and surface precipitation over the inner domain on the 5th of October 2022 at 0900 UTC (created using VAPOR: A Visualization Package Tailored to Analyze Simulation Data in Earth System Science v 3.6.0 http://www.vapor.ucar.edu/).

It is observed that the rainfall over the study area was mediated by warm and cold cloud processes. In fact, LIDAR observations also indicated mixed-phase clouds extending up to 15 $\mathrm{km}$ over the region for this day (https://www-calipso.larc.nasa.gov/products/lidar/browse_images/exp_index.php). A 3D rendering of the cloud volume and the resulting accumulated surface precipitation on the 6th of October 2022, shown in Fig. 11, indicates more rainfall over the Kollidam stretch than in the Kallanai region, as also reported^2,3^. The north-westerly wind shear is also evident from this figure.

**Figure 12:**
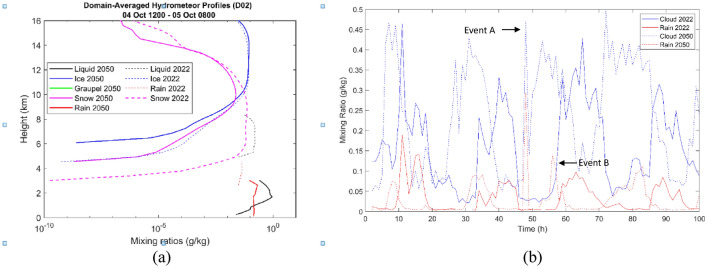
(**a**) Hydrometeor profiles for present and future scenarios. (**b**) Temporal evolution of domain-averaged hydrometeor mixing ratios in the inner domain for the present and future scenarios. Note the many instances when the cloud amount is higher in the 2050 scenario than in the 2022 case. One also notices higher peak rain amounts over short durations in the 2050 case implying an increased probability of flash floods over the region in question (created using MATLAB R2022a (Academic License) https://www.mathworks.com).

Figure 12a and b respectively show the comparison of the present-day with a future scenario. *The peak mixing ratios for liquid cloud and rain in the future scenario are up to an order of magnitude higher than the present scenario*. With such high cloud mixing ratios, it is anticipated that the rain amounts will also be greater than the present scenario as expected (see Fig. 12b), indicating that flood alerts will have to be issued with precision in the future. Additionally, with such displaced rain amounts cropping patterns will have to be recalibrated over the Kaveri delta.

A cross-sectional plot with the floodwater flow path is presented in Fig. S4. The boundary geometry for the analysis is specified in terms of the ground surface profiles, i.e. cross sections, which are perpendicular to the flow lines and are located at specified intervals along a stream flow to ascertain the flow-carrying capacity of the stream and the adjacent floodplains.

**Figure 13:**
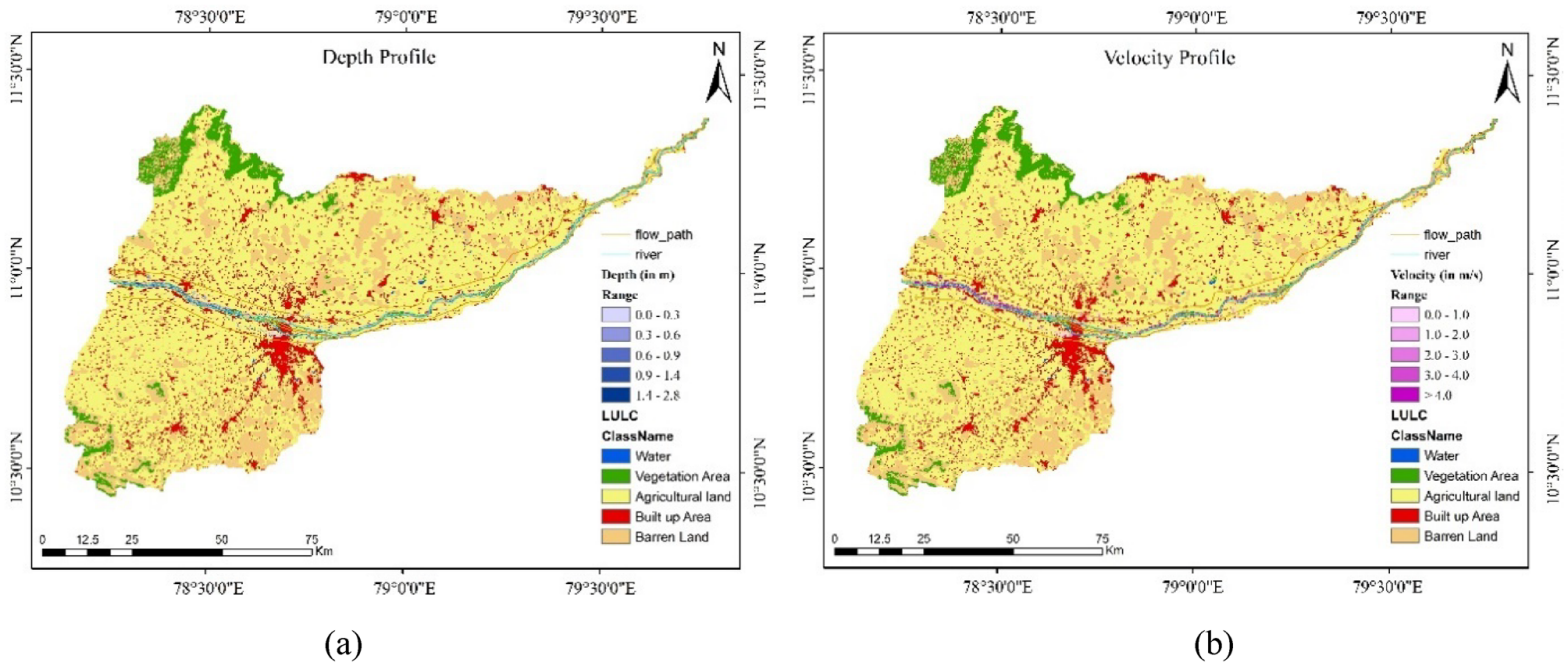
(**a**) Map of the depth profile of the flood-prone area along with LULC. (**b**) Map of the velocity.

To obtain maps of depth and velocity profiles it was necessary to feed outputs from the HEC-RAS to ArcGIS software. From the Fig. 13a and b one observes that both the Kollidam as well as the Kaveri rivers were inundated. The total extent of submergence along riverbanks and other flow paths was 145.98 ${\mathrm{km}}^{2}$ out of which 65.14% of the submerged area was agricultural land. The percentages comprising barren land, built-up areas and vegetated areas were 21.47%, 11.64% and 1.74%, respectively. It is disconcerting that the percentage of built-up areas was quite high with encroachments nibbling away precious vegetated areas over several decades. This aggravates flood risks and as was pointed out in the introduction; emergency evacuation was necessary to move people away to safer zones recently.

profile of the flood-prone area along with LULC (created using ArcGIS, Esri^42^ https://www.arcgis.com/index.html).

**Figure 14:**
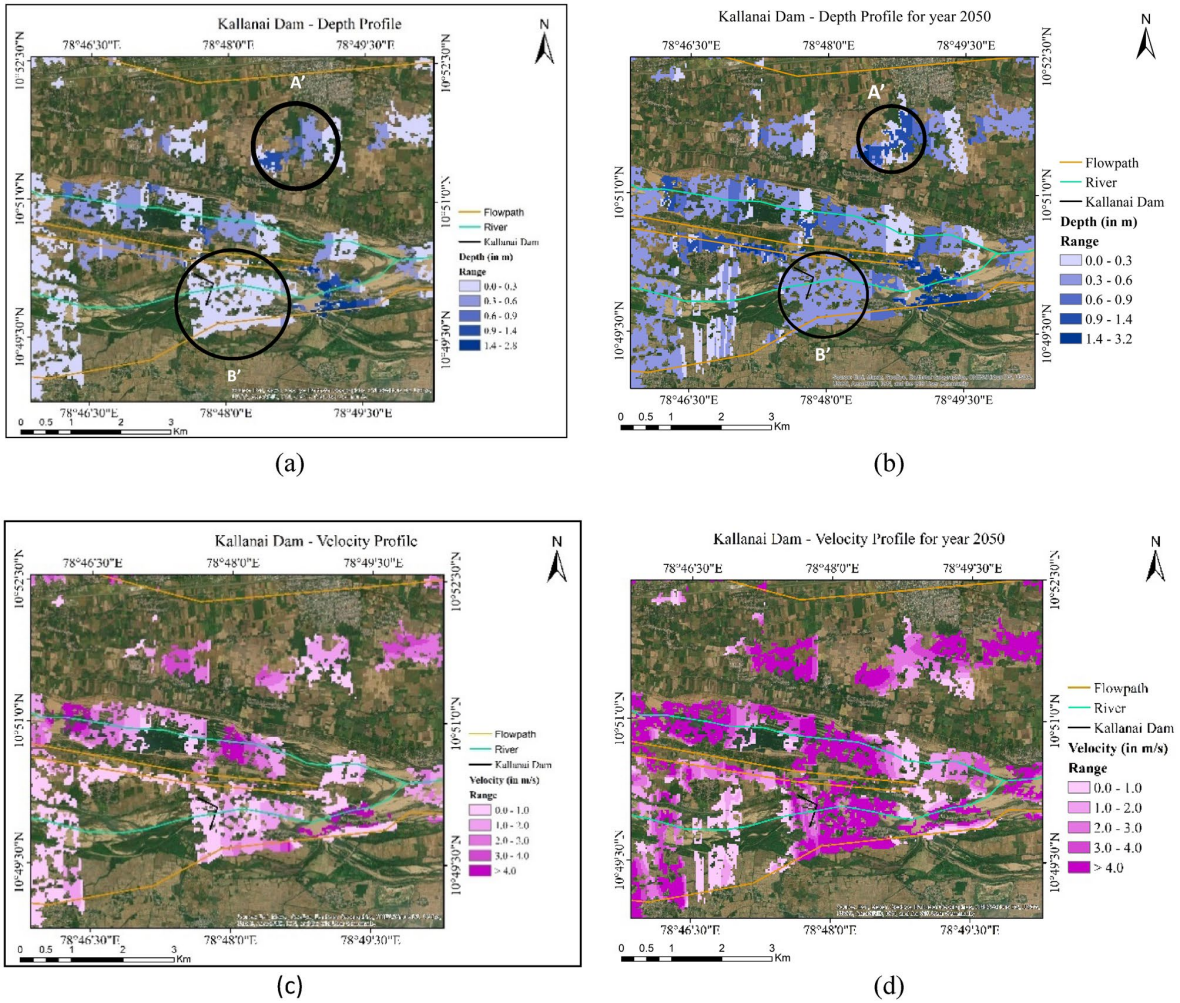
(**a**) Depth Profile in inundated regions around the Kallanai Dam for the 2022 case. (**b**) Depth Profile in inundated regions around the Kallanai Dam for the 2050 case. (**c**) Velocity Profile around the Kallanai Dam for the 2022 case. (**d**) Velocity Profile around the Kallanai Dam for the 2050 case (created using ArcGIS, Esri^42^ https://www.arcgis.com/index.html).

This study yielded well-marked floodwater depth and velocity profiles over the study region (Fig. 14a–d) for the present and future scenarios. *On comparing, *Fig. 14*a and b, one notices that the spatial coverage of floodwater is higher in the RCP8.5 case with greater coverage of blue zones indicating up to two times more inundation than in the present scenario*. This is evidenced by the change in the colour gradients in zones $A{\prime}$ and $B{\prime}$ from light blue (up to 0.3 $\mathrm{m}$) in the 2022 case to dark blue (0.6 $\mathrm{m}$ and above) in the 2050 case. Also, one must contrast the absence of light blue regions along the upstream side of the Kallanai dam during 2022 with them being present during 2050 indicating several zones prone to flash floods in future warming scenarios. The estimated floodwater depths for these two cases must be discussed in context with the guidelines issued by the National Centre for Coastal Research, Government of India^46^. A red atlas was developed as a ready reckoner for flood disaster mitigation wherein regions with floodwater depth in the range of 0.3–0.6 $\mathrm{m}$ are classed as 75% vulnerable to rapid submergence. High inundation depths seen during 2050 indicate greater flood vulnerability of inhabited localities over these regions.

The velocity profile map for the 2022 run shown in Fig. 14c reveals that the speed of flowing water in the inundated regions sometimes exceeds 3 $\mathrm{m}/\mathrm{s}$ over a few areas close to the dam sites. This high-velocity flow can erode agricultural land resulting in soil loss and decreased fertility. Further, such high-velocity water flow can inflict physical damage to structures, particularly those positioned close to the riverbanks. The impact of high-velocity water can be particularly severe during major flooding events when the water flow velocity can even exceed 4 $\mathrm{m}/\mathrm{s}$ (magenta region) as observed from the above figures. It is anticipated that these threats are likely to be aggravated during 2050 and one confirms this with a comparison of the velocity profiles in the two cases. It is observed from Fig. 14d that the velocity heads in the flooded regions during the future 2050 case are likely to be *higher by up to two times* (note the change in the colour gradients from pale pink (up to 2 $\mathrm{m}/\mathrm{s}$) in the 2022 case to magenta (3–4 $\mathrm{m}/\mathrm{s}$ and above) in the 2050 case) indicating clear submergence of all low-lying villages around the major watersheds, i.e. SW3 and SW4.

**Figure 15:**
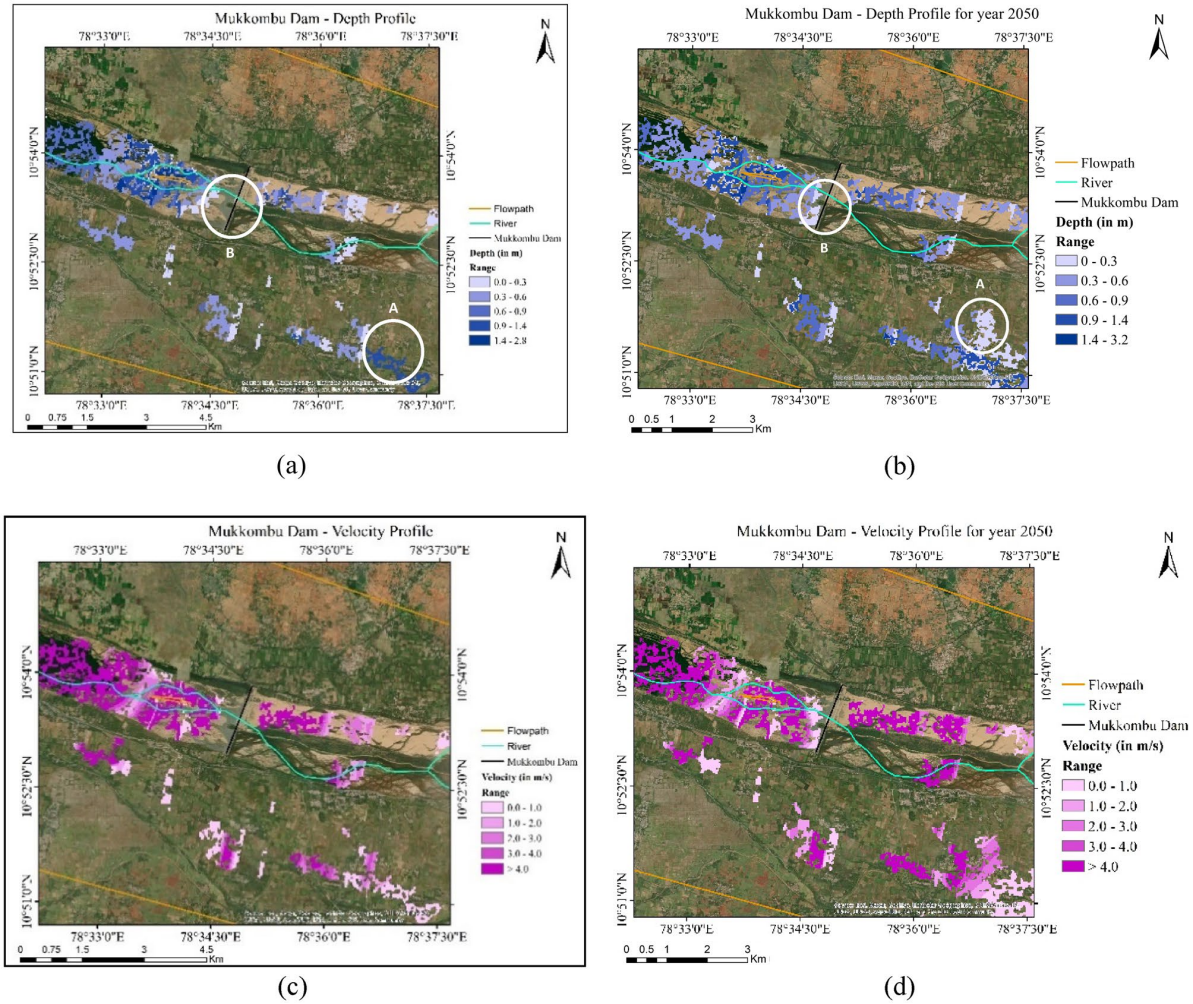
(**a**) Depth Profile in inundated regions around the Mukkombu Dam for the 2022 case. (**b**) Depth Profile in inundated regions around the Mukkombu Dam for the 2050 case. (**c**) Velocity Profile around the Mukkombu Dam for the 2022 case. (**d**) Velocity Profile around the Mukkombu Dam for the 2050 case (created using ArcGIS, Esri^42^ https://www.arcgis.com/index.html).

One notices that upstream regions adjoining the Mukkombu Dam (marked B in Fig. 15) are more prone to flooding during future scenarios with velocity heads approaching 2 $\mathrm{m}/\mathrm{s}$ compared to ‘no’ flooding observed in the 2022 case. Additionally, the South-eastern flank of Mukkombu Dam (marked A in Fig. 15a and b) comprising of built-up areas housing farmers involved in agrarian activities in the adjoining areas is more susceptible to flood-water inundation in the ‘high-emissions’ scenario.

### Threats to water resources over this region

Vulnerabilities associated with flood hazards must be quantified to assess the nature of stability in the region vis-à-vis people's movement through floodwaters and placement of shelters. The degree of hazard varies with the severity of flooding and its hydraulic behaviour, i.e. the velocity heads of water flow in the submerged zones, the extent of inundation, topography, and the population at risk. The potential of floods to cause damage can be indexed against vulnerability curves linked to meaningful hazard thresholds^47^. Hazard potential can be assessed by simply multiplying the level of inundation with the velocity heads in those regions. For instance, the flood hazard potential near the Kallanai dam (Fig. 14) is estimated to be less than or equal to 0.6 in 2022. This varies in the range of 0.9–2.4 during 2050. Whilst in the former 2022 case the Hazard Vulnerability Classification tier is H2 (unsafe for small vehicles), in the latter threat escalates to H5 (Unsafe for vehicles and people where buildings are vulnerable to structural damage, and some may be subject to failure). In fact, certain other regions even attain H6 indicating the highest levels of threat to property and lives. Similarly, over the Mukamboo dam area, the indices are 1.8 (2022) and doubling to 3.6 in 2050 with Hazard Vulnerability Classifications of H4 (Unsafe for vehicles and people) and H5 (Fig. 15). These new results indicate greater flood hazard potentials by up to two times linked to high-intensity precipitation events in future warmed-up scenarios. Results produced from the present analyses can serve as a ready reckoner to support decisions in prioritizing emergency support in vulnerable zones and also enable future planning, i.e. constructing shelter homes and shifting agricultural activities in the least vulnerable areas.

### Optimal policies and management protocols

The problem of undesirable encroachments in the region is already precipitating a humanitarian problem. We have shown through GIS mapping that major watersheds are expected to face grave threats from flooding in the Cauvery and Mukamboo rivers. The Government of India has a periodic dam inspection schedule to reinforce not just the shutters but also the most vulnerable areas of the structure itself. It has been done in the past during the British Raj. A recent Government of India document has provided a ready reckoner in the form of contour maps of a colour-graded vulnerability index^47^. This is a very useful qualitative guideline and we have seen that the hazard potential levels are likely to increase from the current lower tiers now to much higher grades of vulnerability later. This study proposed that government guidelines must be updated periodically to account for the effects of future intense precipitation events. Optimal policies and management protocols must also relate to early-warning systems. It may not always be possible to give sufficient lead times for evacuation during extreme weather occurrences. However, a foreknowledge of the most vulnerable submergence zones will greatly help^48^. This foreknowledge can be assessed through the procedure we have adopted coupling GIS and high-resolution climate modelling with a hydrological model. It is suggested that such results emanating from research institutes are made available through a common web platform accessible to any stakeholder. This is a serious matter for an over-populated country like India largely fed by paddy-cultivation. Crop submergence for prolonged durations results in crop rotting affecting the nation’s GDP^49,50^.

## Wider implications

As short duration but intense precipitation events become the new norm in this era of the Anthropocene, one must assess the impact of such events on vulnerable populations. About 5 million people are engaged in farming as well as fishing with their lives tied up with available water resources in Tamil Nadu’s rice bowl, i.e. the Cauvery delta. The district Revenue Department estimated the flood damages to be about 70 million USD for 2004–2013^51^. Climate change predictions are dire and project an intensification of floods. In a warmer planet rising temperatures will increase crop water demand and exacerbate evaporative losses. It is anticipated that by 2050 there will be a rise in the prevailing maximum temperatures in the range of 1.0–1.5 $^\circ \mathrm{C}$. In this paper, we have for the first time shown the extent of inundation along the flood plains of this region for 2050 and have compared this with the baseline year of 2022 (Figs. 14 and 15). It is found that the extent of flood threats increases by up to two times. Water for irrigation in the Cauvery Delta is sourced from the Cauvery River through the Kallanai Dam or the Grand Anicut, the system of irrigation have evolved over 2000 years with the first dam put in place by the Cholas and lending it a world stature. In the past (and to some extent even now) natural rivers were adapted to function as irrigation canals- the overall result is a network comprising natural and man-made canals and drains. This established order is likely to be severely impacted by short-duration flooding events and might weaken existing embankments catastrophically. We believe that the results from this study will aid policymakers in the Water Resource Department, particularly with regard to providing non-structural interventions. Examples include improved decision-making on water resources, inundation mapping and better-managing flood risks and threats, and also provide simple guidelines for hazard quantification. The latter of course is an entire stand-alone study that we shall undertake in the future.

### Supplementary Information

Supplementary Figures.

## Data Availability

All data generated or analysed during this study are included in this published article [and its supplementary information files].
