# Supplementary material for: The impact of short-duration precipitation events over the historic Cauvery basin: a study on altered water resource patterns and associated threats
Source: Sci Rep. 2023 Aug 29;13:14095. doi: 10.1038/s41598-023-41417-6 (PMC10465519; doi:10.1038/s41598-023-41417-6)
Supplement: Supplementary file 1 — Supplementary Figures. [file 41598_2023_41417_MOESM1_ESM.docx]

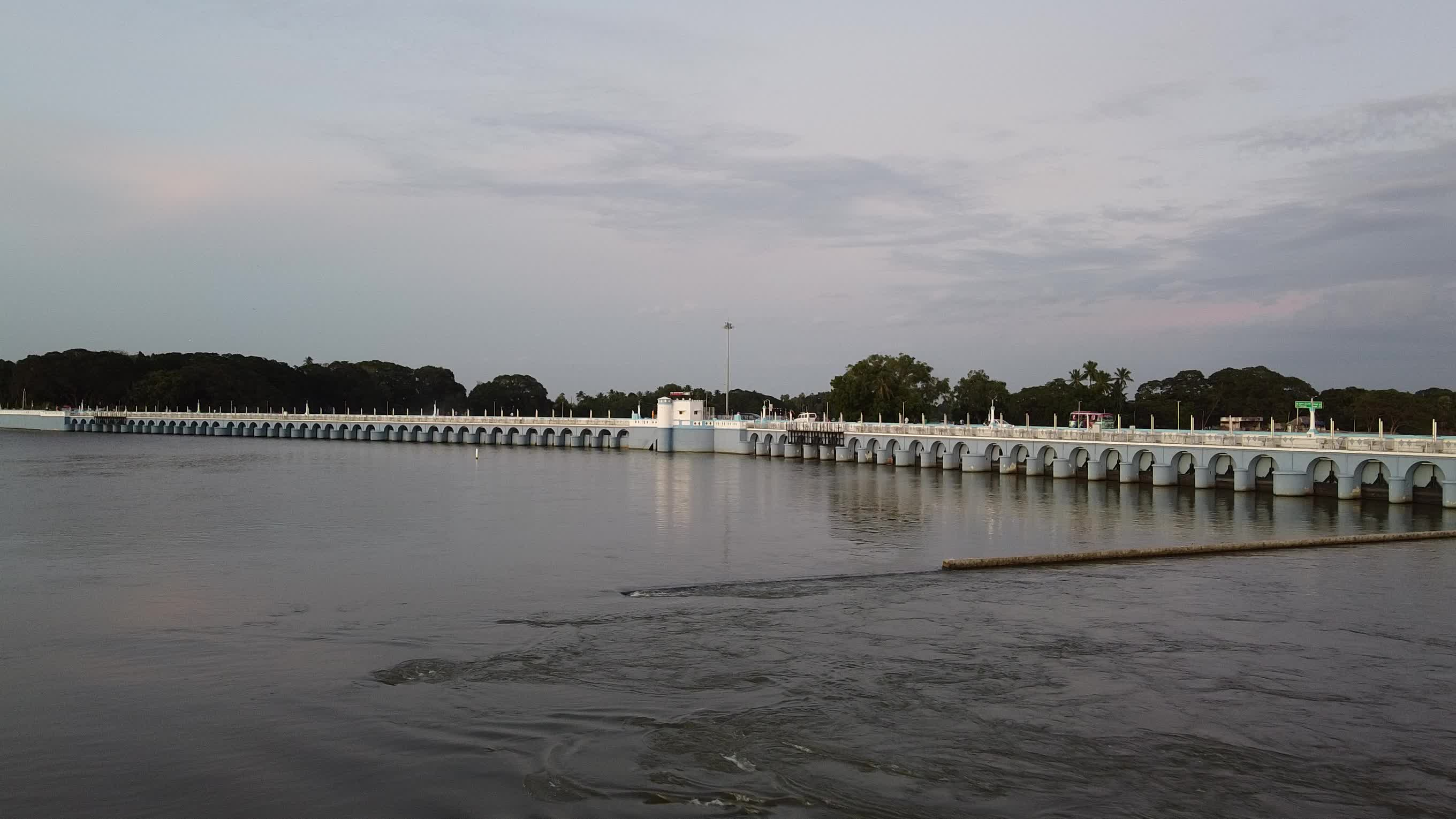


S1: Areal view of flooding along upstream and downstream parts of the Kallanai Dam (Photos: Authors and Arth Chowdhury <https://insidefpv.com/>)


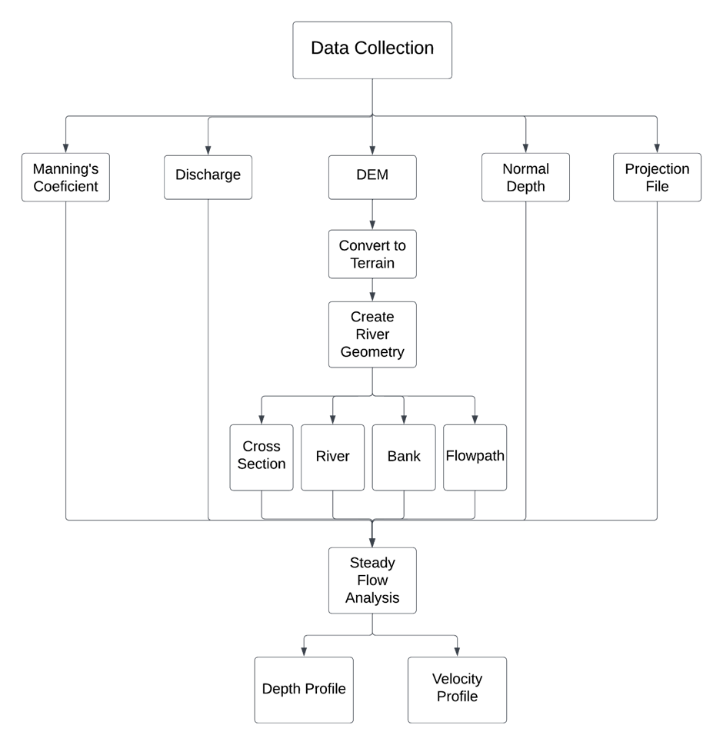


**Figure S2** Methodology for Hydrological Flood Modelling in HEC-RAS

| 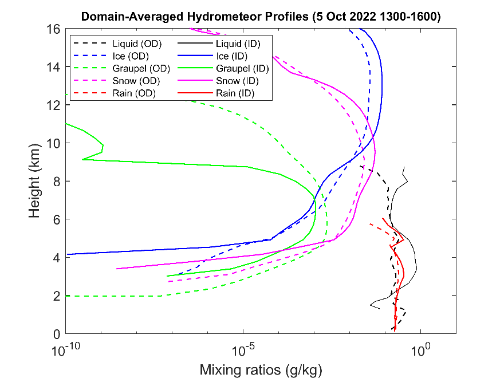 | | 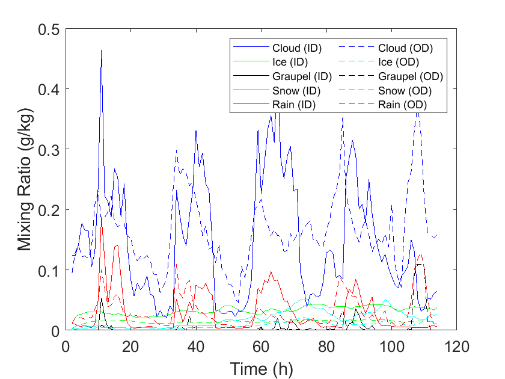 | |
| --- | --- | --- | --- |
| (a) | | (b) |  |

**Figure S3:** (a) Vertical profiles of domain-averaged hydrometeor mixing ratios in the outer and inner domains. The dashed lines correspond to the profiles in the outer domain, whilst the solid lines pertain to the mixing ratio profiles in the inner domain. The cloud and rain amount in the inner domain centred over the Kallanai and Kollidam region is higher than in the larger parent domain covering the entire southern peninsular area (b) Temporal evolution of domain-averaged hydrometeor mixing ratios in the inner and outer domains. Note the many instances when the cloud and rainwater amounts are higher in the inner domain (solid lines) than in the outer domain (dashed lines). An increased liquid cloud also translates to higher rainwater (created using MATLAB R2022a (Academic License) URL: <https://www.mathworks.com>)


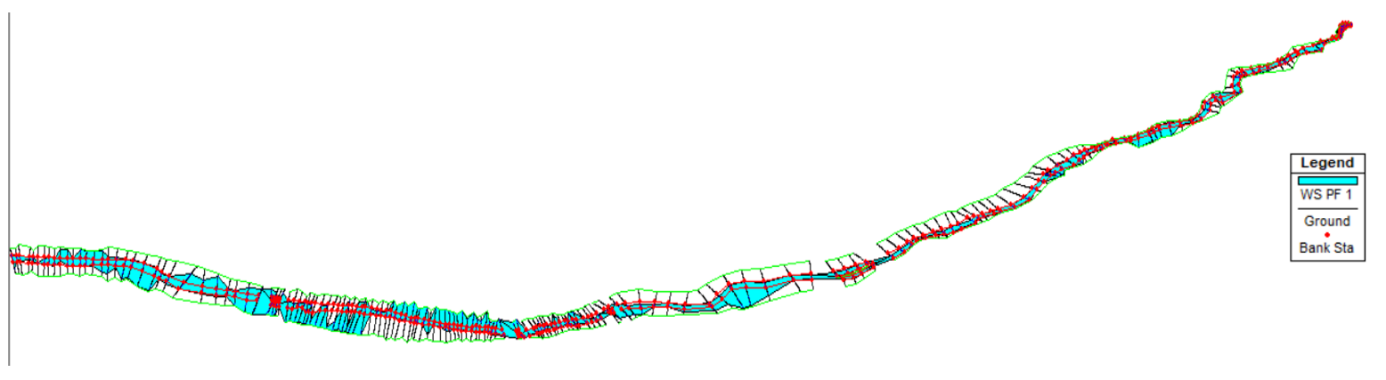


**Figure S4:** Cross-section along the river to ascertain the flow-carrying capacity of the stream and the adjacent floodplains (created using ArcGIS, Esri (2022) URL: <https://www.arcgis.com/index.html>)
